# Recent advances in the repair of degenerative intervertebral disc for preclinical applications

**DOI:** 10.3389/fbioe.2023.1259731

**Published:** 2023-09-22

**Authors:** Yijian Ying, Kaiwen Cai, Xiongxiong Cai, Kai Zhang, Rongzhang Qiu, Guoqiang Jiang, Kefeng Luo

**Affiliations:** ^1^ Health Science Center, Ningbo University, Ningbo, Zhejiang, China; ^2^ Department of Orthopaedics, The First Affiliated Hospital of Ningbo University, Ningbo, Zhejiang, China

**Keywords:** intervertebral disc, fibrous annulus, degeneration, repair, tissue engineering, biomaterials, hydrogel, scaffold

## Abstract

The intervertebral disc (IVD) is a load-bearing, avascular tissue that cushions pressure and increases flexibility in the spine. Under the influence of obesity, injury, and reduced nutrient supply, it develops pathological changes such as fibular annulus (AF) injury, disc herniation, and inflammation, eventually leading to intervertebral disc degeneration (IDD). Lower back pain (LBP) caused by IDD is a severe chronic disorder that severely affects patients’ quality of life and has a substantial socioeconomic impact. Patients may consider surgical treatment after conservative treatment has failed. However, the broken AF cannot be repaired after surgery, and the incidence of re-protrusion and reoccurring pain is high, possibly leading to a degeneration of the adjacent vertebrae. Therefore, effective treatment strategies must be explored to repair and prevent IDD. This paper systematically reviews recent advances in repairing IVD, describes its advantages and shortcomings, and explores the future direction of repair technology.

## 1 Introduction

IDD is frequently asymptomatic (Matsumoto et al., 2010; Brinjikji et al., 2015), but it remains one of the leading causes of LBP (Morlion, 2013; Hartvigsen et al., 2018), a severe chronic disorder that is a significant cause of disability (Hicks et al., 2009; March et al., 2014). It affects approximately 1 billion people worldwide (Hoy et al., 2014). It severely reduces patients’ quality of life while imposing a substantial socioeconomic burden, costing $253 billion per year in the United States alone (Hoy et al., 2014). Current measures to treat LBP due to IDD include conservative and surgical treatment. It is commonly recommended to undergo conservative treatment before surgical treatment, and the choice of treatment depends on the effectiveness of conservative treatment and the degree of IDD (Sabnis and Diwan, 2014; Wu et al., 2020). Conservative treatment includes oral pain medication or topical pain relief, physical therapy and physical exercise to strengthen the low back muscles (Wu et al., 2020). Pain will be relieved in some patients, but recurrence is common and cannot be avoided (Hestbaek et al., 2003). Surgical intervention may be considered when conservative treatment fails to improve symptoms (Storm et al., 2002). There are three main types of surgical treatment: percutaneous decompression, fusion, and disc replacement. Percutaneous decompression includes thermal (radiofrequency ablation), chemical (protease) (Kim et al., 2018) and endoscopic decompression (Aichmair et al., 2014). It relieves the protruding IVD from compressing the nerve root by eliminating the protruding tissue. However, due to the low number of AF cells, avascularity (Hampton et al., 1989; Vernon-Roberts et al., 2007) and poor regenerative capacity (Bailey et al., 2013), there is a high incidence of reherniation and recurrent pain after surgery (Andersson, 1999), which may even lead to degeneration of the adjacent vertebrae (Häkkinen et al., 2007; Hughes et al., 2012). Fusion surgery limits the mobility of the fused segment (Helgeson et al., 2013), while the prosthesis is prone to displacement after total disc replacement (van den Eerenbeemt et al., 2010; Costi et al., 2011). Surgical treatment focuses on relieving pressure to relieve pain and does little to regenerate tissue and delay IDD.

Current strategies for treating IDD include AF sutures and tissue-engineered biological repair. AF sutures maintain IVD height and lumbar stability but do not provide high mechanical loading for long periods. Moreover, suture manipulation is difficult with larger defects (Heuer et al., 2008; Rickers et al., 2018; Li et al., 2020; Ren et al., 2020; Chen et al., 2023). In the last decades, tissue-engineered biological repairs, such as hydrogels and scaffolds, has shown great advantages. However, it still has some limitations at present: 1) Tissue-engineered repair may have a series of safety issues such as implant-induced infections, sterility and tumorigenicity (Agnol et al., 2019); 2) Since the benefit-risk ratio of tissue-engineered repair for human treatment is unknown at present, this will bring a series of ethical issues; 3) The process of tissue-engineered repair is generally long, and it needs to be considered whether it can be repaired efficiently and prevented from recurrence; and 4) At the present stage, the majority of tissue-engineered repair materials mostly remain in the experimental stage, and the treatment of IDD remains a challenge. Hence, this article systematically reviews the recent advances in IVD repair and explores the future direction of IVD repair technology.

## 2 The structure and function of IVD

The IVD is a disc-like fibrocartilage between the vertebral bodies of two adjacent vertebrae consisting of the AF and the nucleus pulposus (NP) (Klein et al., 1983). The peripheral AF wraps around the central NP and anchors within the superior and inferior EPs. The IVD is composed mainly of type I and type II collagen fibres, with a progressive decrease in the distribution of type I collagen from the outside to the inside and the opposite trend for type II collagen. The collagen fibres of the AF make up approximately 70% of its dry weight and the NP approximately 20% (Eyre and Muir, 1976; Eyre and Muir, 1977). The AF is a laminar complex of 15–25 concentric circles (Humzah and Soames, 1988; Marchand and Ahmed, 1990). The angle of the AF fibres varies and cross-aligns between 62° in the outer layer and 20° in the inner layer (Cassidy et al., 1989). The cellular distribution of AF varies, with the cells in the outer annulus showing a tendency to be spindle-shaped fibroblast-like and the inner annulus tending to be oval or spherical (Errington et al., 1998; Bruehlmann et al., 2002). The NP is a heterogeneous structure consisting of glycosaminoglycan (GAG), type II collagen fibres and a small number of chondrocytes (Humzah and Soames, 1988; Mizuno et al., 2004). NP cells are derived from notochordal cells (Choi et al., 2008). The high water content of NP tissue allows it to absorb spinal loads and transmit them to the surrounding tissues (Chan et al., 2011). Endplate (EP) is a layer of hyaline cartilage approximately 0.6 mm thick (Raj, 2008). The cell density in EP is higher than in AF and NP (Maroudas et al., 1975). EP forms a porous, semi-permeable barrier between the vertebrae, regulating the transport of nutrients, mainly small solutes (Chan et al., 2011; Pattappa et al., 2012). IVD loading mechanisms are complex and allow the spine to produce movements such as extension, flexion, bending and rotation (Roberts et al., 2006). IVD features nonlinear, viscoelastic and anisotropic mechanics (Elliott and Setton, 2001), contributing to the spine’s ability to increase flexibility and cushion pressure (O'Connell et al., 2009).

## 3 Pathophysiology of IDD

The pathophysiology of IDD is complex, and the main alterations include loss of proteoglycans, reduction of collagen fibrils and increase in fibronectin (Oegema et al., 2000). The most significant of these alterations is the loss of proteoglycans. The major influential factors are smoking, obesity (Kim et al., 2020), injury (Osti et al., 1990), abnormal mechanical loading (Lotz et al., 1998), genetic susceptibility (Heikkilä et al., 1989), and reduction in nutrient supply to IVD cells (Nachemson et al., 1970). As age increases, the supply of nutrients to IVD cells decreases, decreasing the oxygen content, pH and extracellular matrix (ECM) synthesis of the IVD (Ishihara and Urban, 1999). However, the reduced ECM synthesis would exacerbate the decline in nutrient supply to the IVD (Kim et al., 2020). Ultimately, it causes various pathological changes such as height loss of IVD, vertebral osteophyte formation, EP sclerosis, AF injury, disc herniation, inflammation and motion disorders (Lotz and Ulrich, 2006; Guterl et al., 2013; Huang et al., 2013).

## 4 Hydrogel repair strategy

Hydrogel is a class of materials with a polymer network system capable of absorbing vast amounts of fluid. It is capable of mimicking the biological, chemical and mechanical properties of native tissues. Notably, the mechanical and biological properties and degradation kinetics of hydrogels can be customised to meet different demands (Gauvin et al., 2012; Lewns et al., 2023).

### 4.1 Synthetic hydrogels

Synthetic hydrogels include polyacrylic acid and its derivatives, polyethene oxide, derived copolymers, polyvinyl alcohol (PVA), poly-vinyl pyrrolidone (PVP), polyethylene glycol (PEG) and polyphosphazene nitrile (Yan et al., 2021). Table 1 outlines recent novel strategies for synthetic hydrogels in IVD repair. The biodegradable electrospun polycaprolactone (PCL) membranes show no mass loss or dimensional change after 6 months of exposure to aqueous solutions (Alexeev et al., 2021). Agnol et al. made a polyurethane (PU) tissue adhesive-U2000-2 prepolymer by combining polycarbonate diol and hexamethylene diisocyanate (HDI) monomers. It features excellent dynamic compression properties and adhesion, especially for covalent binding to gelatine without the use of catalysts. However, sterility, biomechanics of *in vivo* and biocompatibility are yet to be investigated (Agnol et al., 2019). HDI in copolymers of PEG with trimethylene carbonate 3 (TMC3) and HDI end-groups is capable of forming covalent bonds with natural AF tissue. At the same time, TMC3 has properties such as high adhesion strength, slow degradation, and excellent cytocompatibility, but extrusion occurs under high stress and cyclic loading, requiring further optimization of TMC (Long et al., 2018). Schmitz et al. reported that PEG combined decellularisation of notochordal cell derived matrix (DNCM) showed excellent biocompatibility, but further biomechanical adjustments are required (Schmitz et al., 2023). Regarding the progress of research on PVA hydrogel as an NP replacement material, Leone et al. found that PVA with PVP molar ratio of 1:1 has excellent biomechanical characteristics which is a promising NP replacement material (Leone et al., 2019). Permana et al. reported that the biomechanical properties of PVA combined with Silicone Rubber close to human NP (Permana et al., 2022).

**TABLE 1:** Synthetic hydrogels repair strategies.

Material	Component	Advantages	Drawbacks	Type of model	Refs
Biodegradable electrospun PCL membrane	Polycaprolactone (PCL)	Slow degradability Long-term repair	—	*In vitro*	Alexeev et al. (2020)
U2000-2 prepolymer	Polycarbonatediol; Hexamethylene diisocyanate monomers	Excellent adhesion; Covalent binding with gelatin without the catalyst	Sterility Biomechanics and biocompa-tibility unknown	*In vitro*	Agnol et al. (2019)
Copolymers of TMC and HDI	Polyetheneglycol; TMC; HDI	Excellent adhesion and cytocompatibility	Poor mechanics	*In vitro*	Long et al. (2018)
PEG combined with DNCM	PEG; DNCM	Excellent biocompatibility	Require further biomechanical adjustment	*In vitro*	Schmitz et al. (2023)
PVA/PVP hydrogel	PVA; PVP	Excellent biomechanics	—	*In vitro*	Leone et al. (2019)
PVA combined with Silicone Rubber	PVA; Silicone Rubber	Biomechanical properties close to human NP	—	*In vitro*	Permana et al. (2022)

### 4.2 Natural hydrogels

Natural hydrogels commonly originate from animal and plant extracts, many of which are essential components of human tissues and organs. Its non-toxic and excellent biosafety, biocompatibility and injectability make it widely used for tissue regeneration and repair. The main components include gelatine, collagen, fibrin, hyaluronate, alginate and chitosan. Table 2 overviews recent strategies for natural hydrogels in IVD repair.

**TABLE 2:** Natural hydrogels repair strategies.

Material	Component	Advantages	Drawbacks	Type of model	Refs
Novel temperature-sensitive chitosan hydrogel	Chitosan; sodium bicarbonate 0.075/β-glycerophosphate 0.1	Mechanical properties resembling human IVD; Preserve NP cell activity; Promote the production of GAG	—	*In vitro*	Alinejad et al. (2019)
Nanostructured gelatin colloidal hydrogels	Gelatin	Shear-thinning; Self-healing; Adjustable mechanical properties; Preserve cell activity Support NP-like differentiation of BMSCs	—	*In vitro*	Wang et al. (2021a)
High-density collagen gels	Collagen	Maintain the water content of the NP	Require a longer follow-up period Lack of biomechanical testing	*In vivo*	Moriguchi et al. (2018)
ChABC-treated collagen gels	Collagen	Enhance adhesion	Safety needs to be clinically tested	*In vitro*	Jiang et al. (2019)
Porcine fibrin gel	Fibrin	Maintain the water content of the NP	Lack of molecular level studies	*In vitro*	Du et al. (2020)
Decellularized ultra-purified alginate gel	Alginate	Facilitate the generation of ECM; Proliferate endogenous NP progenitor cells	Absence of safety tests for immunogenicity; Less suitable for the elderly	*In vivo*	Ura et al. (2021), Tsujimoto et al. (2018)

A novel temperature-sensitive chitosan hydrogel exhibits mechanical properties similar to those of human IVD with the ability to maintain NP cell activity and promote GAG production (Alinejad et al., 2019). Nanostructured gelatin colloidal hydrogels are characterised by shear thinning, self-healing behaviour, homogeneous porous networks and tunable mechanical properties. Notably, it can also prevent leakage of bone marrow stem cells (BMSCs), maintain cell viability, and support the NP-like differentiation of BMSCs (Wang et al., 2021b). The injectable high-density collagen (HDC) gel reduced degeneration of the NP by alleviating AF breakdown and forming a fibrous cap structure that prevented protrusion of the NP contents and maintained the water content of the NP. However, Moriguchi et al. concluded that the long-term effects of the gel are unknown and that it would need to further research analyses and a longer follow-up period (Moriguchi et al., 2018). Jiang et al. used chondroitinase ABC (ChABC) to improve collagen gel adhesion in a short-term manner by removing proteoglycan locally from AF, which reduced the risk of material migration. Moreover, short-term use has not significantly reduced cell viability (Jiang et al., 2019). Porcine fibrin gel was effective in enhancing the closure of AF sutures, helping maintain the NP’s water content and delaying IDD. However, this study needed more evidence at the molecular level (Du and Zhu, 2019).

Alginate is a naturally occurring polymeric polysaccharide found in algal and bacterial cell walls, with high porosity and adjustable viscosity. Decellularised ultrapure alginate (UPAL) gel promotes ECM production and proliferation of endogenous NP progenitor cells. Notably, it also inhibits TNF-α and IL-6 production, downregulates TrkA expression, inhibits vascular endothelial cell degeneration, and reduces acute postoperative pain (Ura et al., 2021). However, the UPAL gel lacks safety tests for immunogenicity, and there is a risk of re-protrusion on the other side of the IVD after injection. It is not well suited to the elderly population owing to its decellularisation (Tsujimoto et al., 2018). Future studies need to refine immunogenicity testing to assess the body’s rejection of it and incorporate stem cell repair to adapt it to the elderly population.

### 4.3 Composite hydrogel

Composite hydrogels are composed of both synthetic and natural polymer materials. Synthetic or natural hydrogels are not able to satisfy both excellent biomechanics and biocompatibility, so in recent years more and more research has turned to composite hydrogels. Table 3 outlines recent strategies for composite hydrogels in IVD repair.

**TABLE 3:** Composite hydrogels repair strategies.

Material	Component	Advantages	Drawbacks	Type of model	Refs
FibGen	Genipin; Fibrin	Better axial compression performance; Improved tensile stiffness and axial neutral zone length of IVDs; Restoration of the water content of IVDs; Delivery of biological factors and cells	Inability to restore axial and torsional parameters to normal levels	*In vitro*	Alexeev et al. (2021), Scheibler et al. (2018), Fujii et al. (2020), Cruz et al. (2018), Frauchiger et al. (2018), Panebianco et al. (2020)
Conjunction of FibGen and CMC-MC	Genipin; Fibrin; CMC-MC	Recover the biomechanics of IVDs; Encapsulate cells and decrease cell leakage	Only axial and torsional mechanics were tested	*In vitro*	Hom et al. (2019)
g-DAF-G	Genipin; Decellularized annulus fibrosus	Differentiate human bone marrow stem cells (BMSCs) to AF cells; Excellent bioactivity and cellular extensibility; Restore the water content of the NP	The method of induced regeneration is inconvenient and not very operable	*In vitro*	Peng et al. (2020)
HA injection combined with photocrosslinked collagen patch	HA photocrosslinked collagen patch	Recover the biomechanics and the hydration of IVDs; Rapidly translate into the clinic	Functional analysis is not focused on the kinematics of the entire spine	*In vivo*	Sloan et al. (2020)
PCL-Supported Electrocompacted Aligned Collagen Type-I Patch	PCL; Collagen	Recover the biomechanics of IVDs Produce sufficient type I collagen and GAG; Promote the adequate proliferation of AF cells	—	*In vitro*	Dewle et al. (2021)
Injectable interpenetrating network hydrogels	Fibronectin-conjugated fibrin; Poly (ethylene glycol) diacrylate; Doubly modified GAG	Higher lap shear adhesive strength than riboflavin cross-linked and Genipin cross-linked fibronectin hydrogels; Non-cytotoxic; Easily apply to AF defects in a short time	Immune responses, changes in pain behavior, *in vivo* degradation, endogenous cellular repair were not assessed	*In vitro*	DiStefano et al. (2020)
Cellulose Nanofiber-Reinforced Chitosan Hydrogel	Cellulose nanofibers; Chitosan	Enhance the mechanical properties; The range of activity of IVD is close to natural IVD	Non cellular repair	*In vitro*	Doench et al. (2019)
Nanocomposite hydrogel	Methacrylated gellan-gum; Cellulose nanocrystals	Enhance the mechanical properties; Mimick the structure of the natural AF interior and exterior; Self-gel	—	*In vitro*	Pereira et al. (2018)
Injectable photocurable hydrogel	PEGDA; (DAFM)	Maintain its porous structure; Promote ECM deposition	Lack of specific markers for AF cells	*In vivo*	Wei et al. (2023)

#### 4.3.1 Restoration and improvement of IVD biomechanics

Genipin is an excellent natural biological cross-linking agent. Genipin cross-linked fibrin (FibGen) hydrogel forms a dense seal at the defect site (Alexeev et al., 2020). It has better axial compression properties than the semi-synthetic binder consisting of glutaraldehyde and albumin (BioGlue) (Scheibler et al., 2018), which improves the tensile stiffness and axial neutral zone length of IVDs. However, it fails to restore the axial and torsional parameters to normal levels (Fujii et al., 2020). Cruz found that FibGen with a ratio of 140 mg/mL fibrinogen and 6 mg/mL genipin (F140G6) provided excellent initial and long-term mechanical properties without cell proliferation and deposition of ECM, making it suitable as a decellularised adhesive and repair biomaterial (Cruz et al., 2018). Notably, FibGen combined with the NP substitute carboxymethylcellulose - methylcellulose (CMC-MC) (Hom et al., 2019), FibGen combined with silk scaffolds (Frauchiger et al., 2018), and injected hyaluronic acid (HA) combined with photo cross-linked collagen patches for repair strategies enhance the mechanical properties of IVD and prevent IDD (Sloan et al., 2020). PCL-supported electrically densified oriented Collagen type I patches have high tensile strength and modulus in the wet state or at sharp increases in stress values (Dewle et al., 2021).

Polysaccharides have various application values such as immune regulation, antiviral, anticancer, and hypoglycemic effects. GAGs doubly modified by oxidation and methacrylation can covalently bind interpenetrating network (IPN) hydrogels composed of fibronectin and polyethene glycol diacrylate (PEGDA) to natural AF tissue, which produces a higher lap shear bond strength than riboflavin cross-linked and genipin cross-linked fibronectin hydrogels. In particular, the degree of oxidation correlates more closely with the adhesion strength than methacrylation. Notably, it is not cytotoxic (DiStefano et al., 2020). The mechanical properties of cellulose nanofibre-reinforced chitosan hydrogels were enhanced due to the addition of cellulose nanofibres. In particular, the range of activity of the IVD after implantation is close to natural IVD. Nevertheless, because of its non-cellular repair, it fails to ensure that the tissue regenerates in the long term (Doench et al., 2019). Gellan gels are polymeric linear polysaccharides widely used in various applications such as food and pharmaceuticals. Pereira et al. reinforced methacrylated gellan-gum (GGMA) with cellulose nanocrystals (nCell), which enhance the ability of AF to withstand loading and create gradient structures that mimic the internal and external structure of natural AF (Pereira et al., 2018). A photocurable hydrogel consisting of PEGDA and decellularised annular fibrous matrix (DAFM) improves the lack of the DAFM hydrogel’s mechanical strength and maintains the porous structure (Wei et al., 2023).

#### 4.3.2 Restore the functionality of NP and AF

The repair strategy of FibGen and injected HA combined with photo cross-linked collagen patches restores hydration and pressurization of IVD, and the latter delivers cells to the NP (Fujii et al., 2020; Sloan et al., 2020). FinGen with a ratio of 70 mg/mL fibrinogen and 1 mg/mL genipin (F70G1) produces ECM at the early stage (Cruz et al., 2018). Low concentrations of FibGen are effective in both delivering biofactors to the injured IVD to enhance endogenous repair and delivering live cells capable of synthesizing ECM for exogenous repair (Panebianco et al., 2020). The repair strategies of photocurable hydrogel consisting of PEGDA and DAFM and PCL-supported electrically dense directed collagen type I patches promote the deposition of ECM and prevent NP atrophy. The latter repair strategy also enables the adequate proliferation of AF cells while providing sufficient ligands for cell attachment (Dewle et al., 2021; Wei et al., 2023).

The repair strategy of PCL-supported electrically dense directed collagen type I patches enables the adequate proliferation of AF cells while providing sufficient ligands for cell attachment (Dewle et al., 2021). Genipin cross-linked decellularised fibrillar ring hydrogel (g-DAF-G) enables the directed differentiation of BMSCs towards AF cells and possesses excellent bioactivity and cell stretching properties. DAF retains the natural microstructure, which reduces the risk of implant rejection (Peng et al., 2020).

## 5 Tissue engineering scaffold repair strategy

Tissue engineering repair is a new therapeutic strategy for repairing IDD using seed cells as the core, scaffolds as the support vehicle and bioactive factors as a facilitating adjunct. Table 4 outlines recent strategies for tissue-engineered scaffolds in IVD repair.

**TABLE 4:** Tissue engineering scaffold repair strategies.

Material	Component	Advantages	Drawbacks	Type of model	Refs
Novel hybrid scaffold of PCL and PLLA	PCL; PLLA	Provide adequate and long-term mechanical support; close to native IVD in terms of tensile properties and cellular response activity; save cost	ECM secretion and scaffold degradation are required to further studies	*In vitro*	Shamsah et al. (2019)
PCL scaffold with angular layer structure	PCL	Excellent elastic response, radial tensile modulus and axial compressibility	Its performance under torsional and fatigue conditions is unknown	*In vitro*	Christiani et al. (2019)
Cell-free biodegradable electrospin PCL scaffold	PCL	Mimic the lamellar structure of natural AF; Excellent mechanical properties; Promote cell colonization, proliferation and organization	Further studies are required to determine the long-term effects of maintaining the structural and mechanical integrity of the IVD.	*In vivo*	Gluais et al. (2019)
Multilayer nanomicrofiber bionic biodegradable scaffold	PCL	Laminar structure resembling native AF; Recover the volume of the NP and slow down the IDD	Scaffold degradation are required to further studies	*In vivo*	Kang et al. (2018)
Electrospin PLLA fibre scaffold	PLLA	Mimic natural AF tissues; Cell morphology, ECM gene expression and protein production resemble native AF tissues	Adjustment of fiber diameter and direction alone is not sufficient, it requires multi-factor adjustment	*In vitro*	Zhou et al. (2021)
Bionic artificial scaffold	PLA; GG/PEGDAPLLA/POSS-(PLLA)_8_	Maintain the IVD height; facilitate proteoglycan deposition; fosters the transfer of cellular nutrients and waste fluid replacement	—	*In vivo*	Zhu et al. (2021)
Aligned nanoyarn scaffold (AYS)	PLLA-PCL Gelatin	Excellent mechanical support	Small pore size	*In vitro*	Wang et al. (2021b)
Nanoyarn/three-dimensional porous nano-fibrous hybrid scaffold	PLLA-PCL	Excellent mechanical support Moderate pore size	The cell infiltration efficiency is low and a significant amount of cells are distributed on the surface of scaffolds	*In vitro*	Ma et al. (2018)
Collagen-PU scaffold	Type I collagen; PU	Excellent cell seeding ability	Its random porous structure is not conducive to matrix deposition	*In vitro*	Du and Zhu (2019)

### 5.1 Mechanical support and imitation of natural AF structures

The mechanical support of the scaffold is important for restoring the biomechanics of the IVD. The fibre angle, diameter and spacing influence the mechanical support of the scaffold (Page and Puttlitz, 2019). A new hybrid scaffold of 50% PCL and 50% poly (L-lactic acid) (PLLA) provides adequate and long-term mechanical support and tensile properties. It is cost-effective compared to 20% PCL and 80% PLLA (Shamsah et al., 2019; Shamsah et al., 2020). PCL scaffolds with an angular layer structure show excellent elastic response and radial tensile modulus, with axial compressibility exceeding natural AF tissue (Christiani et al., 2019).

Mimicking the structure of AF enables scaffolds to exhibit mechanical properties similar to natural AF, restoring NP volume and promoting cell colonisation, cell proliferation and ECM generation (Kang et al., 2018; Gluais et al., 2019). The admixture of GAG in fibrous scaffolds featuring interlaminar structures further enhances the ability of the scaffold to withstand simulated impact loading (Borem et al., 2019). It is insufficient to mimic natural AF tissue by simply regulating fibre diameter and orientation. To better simulate the microenvironment of AF, multifactorial modulation is essential (Zhou et al., 2021). Notably, the biocomposite laminate prepared by Sharabi et al. using alginate hydrogel-embedded long collagen fibres mimics the entire stress-strain mechanical behaviour of the AF lamina in both the longitudinal and circumferential directions, which has great potential for application in IVD repair (Sharabi et al., 2019).

### 5.2 The effect of scaffold aperture size and construction on the repair of IVDs

The porosity and structure of the scaffold are critical for cell migration and diffusion deep into the scaffold, transport of nutrients and metabolites, deposition of ECM and information transfer between cells (Zhu et al., 2021). Although the mechanical support of the nanoyarn scaffold (AYS) is strong, its pore size is small, and the rate of cell diffusion to depth is slow (Wang et al., 2020). The electrospun AYS and nanoyarn/three-dimensional porous nanofibrous hybrid scaffolds (HS) modify the small porosity of AYS, leading to faster cell infiltration efficiency (Ma et al., 2018). The type I collagen-PU scaffold contributes to maintaining the phenotype of human AF cells. However, its random porous structure fails to promote ECM deposition and the formation of a corneal lamellar microstructure similar to natural AF. It would require pretreatment with TGF-β1 to promote cell proliferation and matrix production (Du et al., 2020).

## 6 Several potential repair strategies

Table 5 outlines recent strategies with the potential for IVD restoration.

**TABLE 5:** Additional promising repair strategies.

Strategy	Effects	Defect	Type of model	Refs
ADAM8	Partial inhibition of ADAM8 protein hydrolysis may retard IDD	Only test a small fraction of genes	*In vivo*	Zhang et al. (2021)
Bleomycin	Induction of cellular pro-fibrosis and maintain IVD height; Short-term application of bleomycin would not induce noticeable alterations in the cell cycle and apoptotic rate	—	*In vivo*	Yang et al. (2021)
Low-loading hydrostatic pressure	Low-loading hydrostatic pressure facilitate to maintain cell survival and ECM homeostasis	Bioreactor pressure setting is not precise enough	*In vitro*	Wang et al. (2020)
Low doses of short Link N	Inhibits cell death and promotes IVD synthesis ECM	—	*In vivo*	Mwale et al. (2018)
Autologous PRP	Facilitates early recovery after injury	Need more studies and longer follow-up periods for evaluation	*In vivo*	Gelalis et al. (2019)
Short-term continuous low-tension traction	Provides stable intervertebral environment; Inhibits ECM degradation; Reduces AF tension; Restores IVD height	Excessive traction time lead to IVD degeneration	*In vivo*	Che et al. (2019), Guo et al. (2020)
PBM; Light-guided systems or photosensitizers	Downregulates MMP1 and MMP3 to restrain catabolism	Need more information on additional photoreceptors and effective doses before clinical application	*In vitro*	Hwang et al. (2020)

The strategies of timely and controlled hypotonic traction and photobiomodulation (PBM) improve the microenvironment of IDD by reducing catabolic genes such as MMP-1 and MMP-3 to inhibit ECM degradation, which offers the possibility of non-surgical interventions for patients (Che et al., 2019; Guo et al., 2020; Hwang et al., 2020; Che et al., 2021; Che et al., 2022).

Low hydrostatic pressure facilitates the maintenance of cell survival and ECM homeostasis by upregulating N-cadherin, prominently expressed in NP, and integrin β1, prominently expressed in AF. High hydrostatic pressure triggers apoptosis via the Hippo-YAP/TAZ pathway. Therefore, the repair strategy to keep IVD hydrostatic pressure in a physiologically low load state shows potential (Wang et al., 2021a).

Short-term local injection of bleomycin induces AF cells and BMSCs to promote fibrosis and maintain IVD height through the TGFβ-TGFβR1-Smad2/3 pathway without causing significant changes in cell cycle and apoptosis rates, which preserves the possibility of future clinical application (Yang et al., 2021).

ADAM8 may regulate inflammation and collagen fibril assembly, so partial inhibition of ADAM8 may serve as an intervention to delay IDD (Zhang et al., 2021).

Link N is a glycoprotein consisting of 16 amino acids that stabilize proteoglycan aggregates by combining with HA and integrins. Low doses of short Link N, consisting of one to eight amino acids, inhibit cell death and promote ECM synthesis (Mwale et al., 2018).

The injection of autologous platelet-rich plasma (PRP) within the IVD facilitates early recovery after injury. However, additional studies and more extended follow-up periods are required (Gelalis et al., 2019).

Cell leakage dramatically affects the effectiveness of the repair. Some repair strategies have been shown to reduce cell leakage (Hom et al., 2019; Wang et al., 2021b). Further research on reducing cell leakage is required in the future.

## 7 Currently marketed available IVD repair devices

Suturing of the IVD is a common repair method in clinical practice today. Advantages of AF suturing include 1) maintaining the pressurising effect of the NP and reducing the risk of recurrence of herniation; 2) decreasing mechanical irritation to the nerve root and alleviating postoperative symptoms of low back and leg pain; 3) diminishing the release of inflammatory mediators from the IVD and reducing the incidence of chemical radiculitis; 4) facilitating the healing of the scar in the AF (Bateman et al., 2016; Li et al., 2020). Ahlgren et al. found that sutured sheep IVDs exhibited a greater tendency to heal than non-sutured IVDs (Ahlgren et al., 2000).

**FIGURE 1:**
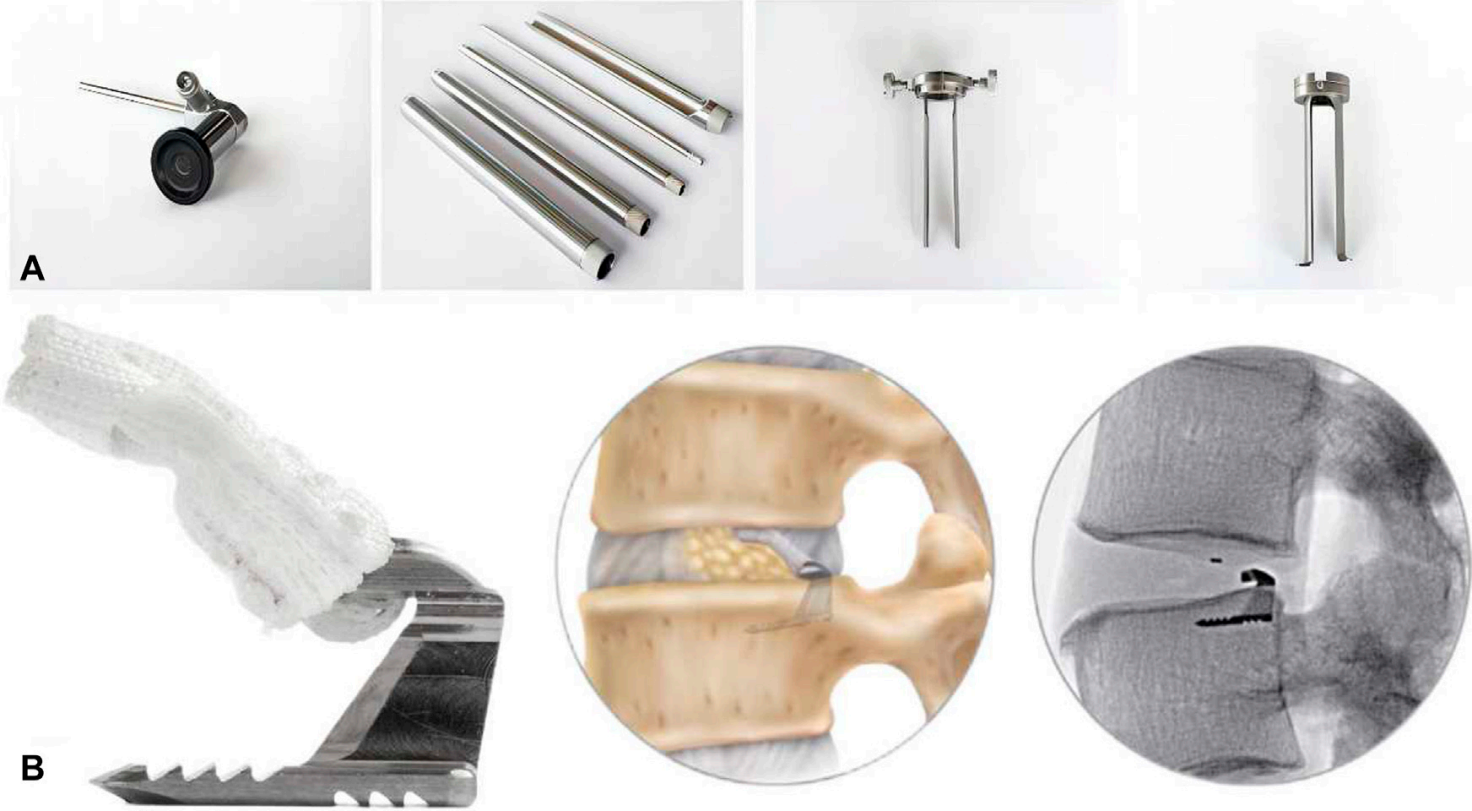
**(A)** The AnchorKnot^®^ Suture-Passing Device. **(B)** The Barricaid^®^ Annular Closure Device and post-implantation simulation.

**FIGURE 2:**
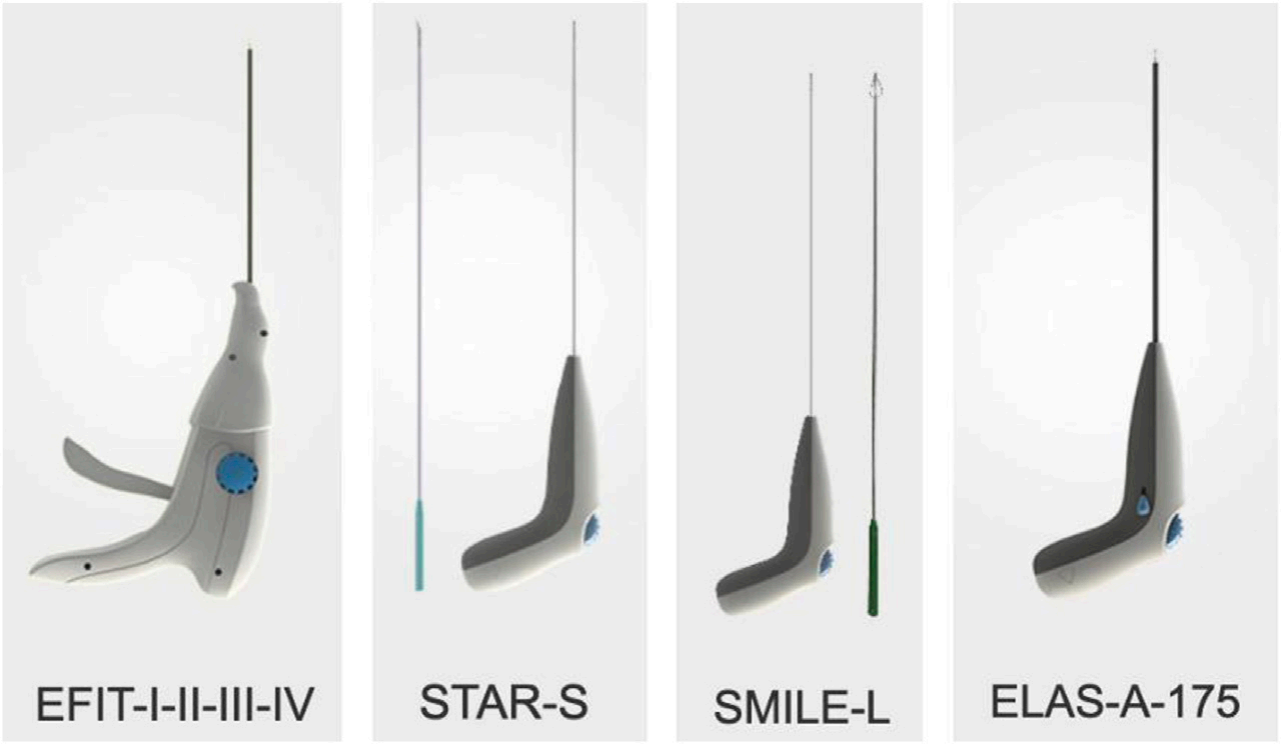
Disposable AF suture device from Beijing 2020 medical science & technology.

In recent years, AF suture repair of IVD has been carried out in clinical practice and has shown promising results. The currently marketed available devices include Beijing 2020 Medical Science & Technology’s Disposable AF Suture Devices (EFIT-I-II-III-IV-V, ELAS-A, SMILE, STAR), The Xclose^®^ Tissue Repair System, The AnchorKnot^®^ Suture-Passing Device and The Barricaid^®^ Annular Closure Device. The devices are shown in Figure 1 and Figure 2. The Xclose^®^ Tissue Repair System reduces the risk of re-protrusion and re-operation, favouring short-term patient outcomes (2 years). It carries no additional risk of surgery. However, postoperative back and leg pain is significant (Bailey et al., 2013; Bartlett et al., 2013; Choy et al., 2018).

The AnchorKnot^®^ Suture-Passing Device allows minimally invasive visualisation of the surgical field and minimises removal of IVD tissue, but safety and efficacy need to be evaluated. The Barricaid^®^ Annular Cloure Device is a titanium body anchored to the adjacent vertebral body that maintains IVD height, reduces pain, decreases reherniation rates and slows the progression of small joint degeneration. However, there are risks of EP fracture, device dislodgement, inflammation, fibrosis, osteolysis, and osteophyte formation (Strenge et al., 2019; Miller et al., 2020; Zengerle et al., 2020; Kienzler et al., 2021; Nunley et al., 2021; Peredo et al., 2021). Parker et al. found that The Barricaid^®^ Annular Closure Device maintained the height of IVD and alleviated symptoms of pain. According to their statistics, the potential savings is approximately $220,000 per 100 surgeries (Parker et al., 2013). A study by Ardeshiri et al. showed that the application of The Barricaid^®^ Annular Closure Device was safe, with a significant reduction in IVD reherniation in postoperative patients (Ardeshiri et al., 2019).

Ren et al. reported that Beijing 2020 Medical Science & Technology’s Disposable AF Suture Devices maintained the height of IVD, relieved postoperative pain and improved function. Its postoperative recurrence rate is lower than that of Percutaneous Transforaminal Endoscopic Discectomy (PTED), but there is no statistically significant difference between them. Table 6 outlines currently marketed available IVD repair devices (Ren et al., 2020).

**TABLE 6:** Currently marketed available IVD repair devices.

Strategy		Effects or scope of application	Defect	Refs
Disposable AF suture device	EFIT-I-II-III-IV	Available under small incisions, dilated channels, Microendoscopic Discectomy (MED)	It is uncertain whether it significantly reduces the recurrence rate of herniation; Need larger sample size	Ren et al. (2019)
	STAR-S			
	SMILE-S			
	ELAS-A-175			
	SMILE-L	Available under large channel spinal endoscopic minimally invasive surgery system and Percutaneous Transforaminal Endoscopic Discectomy (PTED)		
	STAR-L			
	ELAS-A-300			
Xclose^®^ Tissue Repair System		Reduced risk of re-herniation and re-operation (2 years); There is no additional increase in surgical risk	Significant postoperative back and leg pain	Choy et al. (2018), Bartlett et al. (2013), Bailey et al. (2013)
AnchorKnot^®^ suture-passing device		Display the surgical field in minimally invasive fashion; Minimise the removal of IVD tissue	Safety and effectiveness need to be evaluated	Peredo et al. (2021), Miller et al. (2020), Nunley et al. (2021), Kienzler et al. (2021), Strenge et al. (2019), Zengerle et al. (2020)
Barricaid^®^ Annular Closure Device		Maintain IVD height; Relieve pain; Decrease reherniation rates; Slow the progression of small joint degeneration	There are risks of EP fracture, device prolapse, inflammation, fibrosis, osteolysis, and bone redundancy formation	

## 8 Conclusion and outlook

As the pathophysiology of IDD has been increasingly studied in recent years, various repair strategies for IVD have been proposed, including hydrogel repair, tissue-engineered scaffold repair and several promising repair modalities. These therapeutic strategies aim to restore the mechanical properties of IVD, promote cell proliferation and differentiation to repair AF and promote the production of ECM to maintain the water content of NP. However, mechanical properties and biocompatibility are not well satisfied simultaneously, and clinical application is still far off.

Synthetic hydrogel repair strategies are poorly biocompatible, with advantages in terms of mechanical properties. Natural hydrogel repair strategies are poor in mechanical properties, with advantages in maintaining cell activity, promoting cell proliferation and differentiation, promoting ECM synthesis and maintaining the water content of NP. Composite hydrogel repair strategies can improve the biomechanics of IVDs while ensuring biocompatibility, but strategies to enhance mechanical properties still require exploration. In future, hydrogel repair strategies should guarantee biocompatibility with continuous enhancement of mechanical properties or guarantee mechanical properties with continuous enhancement of biocompatibility.

The priority of the scaffold repair strategy is that the scaffold is in place and provides sufficient mechanical strength. Secondly, it is necessary to achieve the appropriate porosity. If the scaffold is less pore size, it is less conducive to cellular penetration, and if the pore size is too large, it is less conducive to mechanical support. Finally, mimicking the AF structure shows excellent potential to exhibit mechanical properties similar to natural AF and facilitate the deposition of ECM.
